# Sporo-Thrichotic Arthritis of the Knee

**Published:** 1910-04-23

**Authors:** 


### Clinical Notes.

### SPORO-THRICHOTIC ARTHRITIS OF THE KNEE.

Sporo-thrichosis of bone has been recorded in man repeatedly; the corresponding affection of joints is rarer, or at least has so far been less often diagnosed, so that the following case is of particular interest. It occurred under the care of Dr. Moure in the Hôtel Dieu, of Paris.

The patient was a man of 40 years of age, a cook by occupation, who had never been severely ill until the month of January 1908. He then began to suffer from cramp-like sensations, and, particularly when he was cold, from irritation in the left leg, especially in the region of the knee, which at the same time began to swell. He received medical treatment and improved in health for a time; but about April 15, 1909, he had to give up his work again and finally was admitted to the hospital; he was found to be suffering from what was regarded as a cold abscess of the upper extremity of the left tibia, together with hydrarthrosis of the left knee. The abscess was punctured and some viscous pus withdrawn; the knee was not punctured, and at this time no bacteriological or cytological investigations were made.

The patient remained in the hospital for six weeks and was then discharged. Some days after his discharge the abscess developed again and the pains in the part returned. A compress saturated with tincture of iodine was applied, and presently the abscess opened spontaneously, a fistula resulting, and within a short time the region around the fistula became ulcerous. A few days after this gumma-like lumps appeared upon the antero-internal aspect of the thigh; the number of these lumps rapidly increased, and the patient was taken to the Hôtel Dieu on January 1. The condition presented by his left leg included ulceration, hydrarthrosis of the knee, and gumma-like masses of the thigh.

### Condition on Examination.

The ulceration was situated over the upper extremity of the left tibia corresponding roughly to the region of its anterior tuberosity. Its surface was covered by yellow-green thick viscous pus; after it had been cleaned up by means of fomentations it appeared red and finely granular. The edges were irregular and undermined, and on pressing the overhanging borders thick pus could be made to exude from beneath them. The surrounding skin for several centimetres round the ulcer was thick and of a bluish-violet colour with a brown border outside this. A little lower down there was a fluctuating tumour which gave one the impression that it was a sub-periosteal abscess.

The knee was uniformly swollen, all the pouches of the synovial membrane and all the ordinary hollows about the joint being filled out. The patella could be pressed down upon the underlying bones, so that there was undoubtedly free fluid present, and when the knee-cap was pressed back against the lower end of the femur a rough kind of grating could be felt which gave one the impression of a surface devoid of articular cartilage. Any pressure upon the knee or upon the upper part of the tibia was painful. From the knee up to the apex of Scarpa's triangle, forming a chain along the course of the saphenous vein, there was a series of twenty-eight subcutaneous nodules, elastic but soft, and varying in volume from that of a small bean to that of a big nut. There was no ulceration over any of these lumps. The skin over them was normal except for some slight brown discoloration over five of them. It was possible to make out that there was lymphatic induration in some places between the nodules.

### Course of the Case.

The inguinal lymphatic glands were slightly increased in volume; they were as large as haricot beans, not painful, not adherent to the skin, firm and fairly movable. A small portion of one of these glands was excised, but the section of it did not present anything definite under the microscope. *X*-ray examination of the region of the knee-joint showed very clearly that the upper extremity of the tibia was much more translucent than normal, particularly at its centre, whilst at the same time there was increase in the density of the bone immediately under the periosteum. The general condition of the patient was not very good, his face was pale, and he had lost weight. His temperature was slightly raised, particularly in the evenings. The lungs seemed to be absolutely normal, and so also did the heart, abdominal organs, nervous system, and urine. The patient had never suffered from either gonorrhœa or syphilis, and there seemed to be no reason to doubt his statement. Owing to the peculiar character of the affection of the knee-joint and its neighbourhood, particular care was taken with the laboratory investigations in this case.

Pus was collected from the surface of the ulcer over the tibia, and it was planted upon tubes of simple gelose, one being incubated at body temperature and the other kept at the ordinary temperature of the room. Both tubes developed numerous colonies of Sporotrichium Beurmanni mixed with some of streptococcus, staphylococcus, and a few of bacillus pyocyaneus.

One of the subcutaneous nodules was punctured with a needle and some thick, yellow, viscous pus was obtained and planted upon tubes containing gelose-glucose and potato respectively. Cultures of the Sporotrichium Beurmanni were obtained from them also, and always earliest and best in the tubes that were incubated at body temperature.

### Treatment.

Treatment by means of 30 grains of iodide of potassium per diem was employed by the mouth, and by successive increase the dose was raised to 120 grains per diem without difficulty. In three weeks' time the subcutaneous nodules and the periosteal lump had disappeared. The ulceration rapidly improved, and at the end of six weeks it was almost skinned over, though there was still a small sinus communicating with the upper end of the tibia. The knee-joint had diminished very greatly in size, and the patient was free from pain. He was discharged a month later, and stopped all treatment, thinking he was well. A relapse resulted, but under a repetition of similar treatment he ultimately became completely cured and remained so.

It will be noted that in the above case the first symptom of the affection was hydrarthrosis, which was apparently causeless. The next changes were in the upper extremity of the tibia, resulting in what seemed to be an obvious cold abscess, so that the patient, originally regarded as syphilitic, was now looked upon as tuberculous. There had certainly been nothing in the case up to this point to rouse any suspicion about the mycosis, and it is on this account that it seems to be of particular interest; for it appears quite likely that there are not a few patients in whom the accurate diagnosis is not arrived at because the discharge or the puncture fluid, as the case may be, is not carefully examined both microscopically and by means of cultures. In the case described it was the series of subcutaneous lumps which first roused suspicion; but even after the spirochæta Beurmanni had been obtained in the liquid from the joint, and in the pus both from the ulcerous discharge and from the subcutaneous nodules, it would not have been safe to affirm that the fungus was really a causal factor, and not an accidental contamination, until accessory means of diagnosis had confirmed it; inoculations into guinea-pigs, however, proved that the lesion was not tuberculous, whereas the positive characters of the serum-agglutination reaction point definitely to the lesion being sporo-thrichotic.

